# A randomized exploratory phase 2 study in patients with chemotherapy-related peripheral neuropathy evaluating whole-body vibration training as adjunct to an integrated program including massage, passive mobilization and physical exercises

**DOI:** 10.1186/s40164-017-0065-6

**Published:** 2017-02-07

**Authors:** Stefan S. Schönsteiner, Heidi Bauder Mißbach, Axel Benner, Silja Mack, Thomas Hamel, Michael Orth, Bernhard Landwehrmeyer, Sigurd D. Süßmuth, Carolin Geitner, Regine Mayer-Steinacker, Anneliese Riester, Andrea Prokein, Elfriede Erhardt, Jelena Kunecki, Anna M. Eisenschink, Rainer Rawer, Hartmut Döhner, Elisabeth Kirchner, Richard F. Schlenk

**Affiliations:** 1 Department of Internal Medicine III, University Hospital Ulm, Albert Einstein Allee 23, 89081 Ulm, Germany; 2 VIV-ARTE®Bewegungsschule, Asselfingen, Germany; 3 German Cancer Research Center Heidelberg, Heidelberg, Germany; 4 Department of Neurology, University Hospital Ulm, Ulm, Germany; 5 Novotec Medical GmbH, Pforzheim, Germany

**Keywords:** Chemotherapy related peripheral neuropathy, Integrated training program, Whole body vibration training, Chemotherapy associated side effects

## Abstract

**Background:**

Chemotherapy-induced polyneuropathy (CIPN) is a common toxicity after chemotherapy, immunomodulatory drugs or proteasome inhibitors, which is difficult to treat and may also have impact on quality of life. The objective of the study was to evaluate whole-body vibration (WBV) on the background of an integrated program (IP) including massage, passive mobilization and physical exercises on CIPN.

**Patients and methods:**

In an exploratory phase-2 study patients with CIPN (NCI CTC grade 2/3) were randomized for WBV plus IP (experimental) to IP alone (standard). 15 training sessions within 15 weeks were intended. As primary endpoint we used chair-rising test (CRT) to assess physical fitness and coordination. In addition, locomotor and neurological tests and self-assessment tools were performed.

**Results:**

A total 131 patients with CIPN were randomized (standard, n = 65; experimental, n = 66). The median age was 60 (range 24–71) years; 44 patients had haematological neoplasms and 87 solid tumors. At baseline, all patients presented with an abnormal CRT. Fifteen (standard) and 22 (experimental) patients left the program due to progression/relapse or concomitant disease. There was no significant difference in the proportion of patients with normal CRT (<10 s) at follow up between experimental (68%) and standard (56%) (p = 0.20). All patients experienced less symptoms and pain (p < 0.001) and had improved CRT (p < 0.001) over time. WBV was significantly associated with a higher reduction of time needed for CRT (p = 0.02) and significantly improved warm-detection-threshold comparing baseline to follow-up assessment (p = 0.02).

**Conclusion:**

Whole-body vibration on the background of an IP may improve physical fitness and coordination in patients suffering from CIPN. *Trial registration* Retrospectively registered at http://www.iscrtn.com (ISRCTN 51361937) and http://www.clinicaltrials.gov (NCT02846844).

**Electronic supplementary material:**

The online version of this article (doi:10.1186/s40164-017-0065-6) contains supplementary material, which is available to authorized users.

## Background

Chemotherapy induced polyneuropathy (CIPN) is a frequent toxicity observed after chemotherapy [1], immunomodulatory drugs or proteasome inhibitors [2], but may also occur with a lower incidence after other novel anti-cancer drugs such as arsenic trioxide [3] or brentuximab [4]. The incidence of CIPN is estimated with 38%, in particular if combination chemotherapy was administered [5]. However the incidence varies depending on chemotherapy-schedule, duration of exposure, and assessment methods [6, 7]. Clinically, CIPN is characterized by sensory loss typically in a symmetrical, distal, “glove and stocking” distribution [8] that can lead to substantial interference with activities of daily life by severely disturbing fine motor skills, pain and insomnia. Furthermore, CIPN may be accompanied by motor and autonomic changes [9]. In a recently published meta-analysis including 4.179 patients a prevalence of CIPN was reported in the first month after chemotherapy of 68%, after 3 months of 60% and of 30% after 6 months [1]. Prevention of CIPN has been studied in several trials but without leading to consistent results in subsequent meta-analyses [10, 11]. Neuroprotective therapeutic approaches using amifostine, glutathione, acetyl-l-carnitine, glutamine, calcium/magnesium infusion, and gabapentin or pregabaline showed no or only little efficacy [12] as did adjuvant therapies with herbal medicine [13]. Treatment with electroacupuncture may be a therapeutic strategy for CIPN in patients with multiple myeloma. However, a randomized study is currently not available to confirm the results of the reported small phase-II study [14].

If CIPN is diagnosed during cancer therapy treatment options are limited; trials using antiepileptic or antidepressant drugs have been negative [15–18]. Recently, one double-blinded randomized trial was positive showing a reduction of chemotherapy-induced peripheral neuropathic pain after 5 weeks of treatment with duloxetine [19].

CIPN heavily affects physical fitness by the severe consequences of loss of peripheral somatosensory information on human balance and locomotion [20, 21]. This observation prompted us to develop a stepwise training program with the aim to improve and specifically train skills necessary for daily life activities [22]. The program was composed of 15 sessions on a biweekly basis of two parts, (i) massage and passive mobilization in posture and transport layers to induce proprioceptive and tactile stimuli and (ii) gymnastics to improve physical fitness. A growing body of evidence indicates improvements of various neuromuscular parameters following whole-body vibration (WBV), such as power, strength, movement velocity, range of motion and in particular balance [23–26]. However, WBV alone did not yet show a clear benefit for patients with peripheral neuropathy [27]. We hypothesized that applying WBV after massage and passive mobilization in posture and transport layers may harness the potential benefits of WBV to patients with CIPN. Therefore, we conducted an up-front randomized explorative phase 2 trial to investigate whether the addition of WBV to our program has the potential to improve outcome in patients with CIPN.

## Methods

### Patients and study design

Written informed consent was obtained from all patients. The protocol was approved by the Ethics Review Committee. Cancer patients between 18 and 70 years of age suffering from solid or hematological neoplasms suffering from CIPN grade II–III according to National Cancer Institute Common Toxicity Criteria (NCI CTC, version 3.0) and pathological chair-rising test (CRT) [28] (≥10 s) were eligible. Exclusion criteria were disability to perform the CRT at all, known psychiatric disorders, plasmatic coagulation disorders, thrombotic/thromboembolic events within 6 months before randomization and severe neurological disorders like seizures. In this single center study, patients were randomly assigned (1:1) to the experimental (including WBV) or the standard arm. Randomization was carried out based on a predefined list with blocks of 4.

### Whole-body vibration platform

The vibration platform Galileo-Fitness [29] (Novotec Medical GmbH, Germany) contains a seesaw plate with adjustable amplitude and frequency fixed at a right angle on an electrically customizable examination couch. The seesaw motion at high frequencies resulted in a high amount of stimulatory impulses to the legs applied during one intervention, similar to the number of impulses received during 2–3 h of walk at regular speed [30].

### Treatment

#### 1st part

All patients received massage and passive mobilization in posture and transport layers [31] for 30 min per side. Joints of the lower extremities were moved passively for warm-up and stretching of muscle groups in their degrees of freedom. The passive mobilization of the legs always started with the most distal joints (toes) moving gradually more proximally.

#### 2nd part

Patients in the experimental arm received training with WBV. All WBV steps were applied according to individual patients’ tolerability. In a warm-up time of 3 min, patients were treated with frequencies starting from 9 Hz and increasing up to 13 Hz in a horizontal position of the examination couch (0° elevation). Afterwards, the position was changed starting with 30° elevation at a frequency of 14 Hz and increasing to an elevation of 60°–90° at a frequency of 18 Hz (3 min). Thereafter, the position was changed in all patients to 90° elevation (up-right position) starting at a frequency of 19 Hz and increased to a frequency of 23 Hz (3 min). Finally, a cool-down phase (9 min) followed with lower frequencies of 9 Hz to 13 Hz decreasing from 30° elevation to a lying position to protect patients from muscle soreness.

Patients randomized to the standard arm switched after part 1 immediately to part 3.

#### 3rd part

Alternating training exercises with a focus on training of posture and transport movements were initiated including 21 separate exercises (Additional file 1: Table S1).

All patients were invited to practice the exercises at home on a daily basis and asked to document their efforts. In addition, all patients were motivated to walk as frequently and long as possible. This was documented by a pedometer (OMRON Step Counter Walking style III; OMRON Healthcare Co., Ltd Kyoto, Japan) during the intervention period. Walking distance was recorded by counting steps cumulatively between the sessions and reported as steps per day.

In total 15 sessions of this program were intended on a biweekly basis.

### Assessment

#### Questionnaires and NCI-CTC scoring

All patients were assessed for severity of peripheral neuropathy before, after 8 and 15 interventions and one month after the last intervention (follow-up) according to NCI-CTC scale by two investigators (S.S. and RFS). In addition, during the same visits all patients were asked to complete the Functional Assessment of Cancer Therapy/Gynecologic Oncology Group neurotoxicity subscale (FACT/GOG-NTX) using the categories “not at all”, “a little bit”, “somewhat”, “quite a bit” and “very much”, and quality of life questionnaire (EORTC QLQ-C30, version 3.0) with composed measures global status, function and symptom score as well as overall QoL [6, 7].

#### Physical examination

The physical examination including neurological assessment was performed before the intervention, after 8 and 15 sessions, as well as at follow up. The examination included patellar tendon reflex and Achilles tendon reflex at both sides, quantitative evaluation of pallesthesia by using a Rydel-Seiffer tuning fork (C64) with a scale from 0/8 (worse) to 8/8 (best), discrimination between cold/warm (Tipterm) and light touch and pinprick, respectively [32].

#### Chair-rising test (CRT)

The test was performed as previously described [29]. Patients were asked to stand up from a standardized chair (height: 46 cm) with their arms crossed in front of the chest for five times as fast as possible. Normal values for the CRT were defined as <10 s. For evaluation both a dichotomized (normal, abnormal) read-out as well as total time needed to complete the CRT in seconds were assessed.

#### Quantitative sensory testing (QST)

The quantitative sensory testing followed a standardized protocol [33]. Patient’s right foot was the test area and the face the reference area. This included the evaluation of the cold detection threshold (CDT), the warm detection threshold (WDT), the thermal sensory limen (TSL), the cold pain threshold (CPT), the heat pain threshold (HPT), the pressure pain threshold (PPT), the mechanical pain threshold (MPT), the mechanical pain sensitivity (MPS), the wind-up ratio (WUR), the mechanical detection threshold (MDT) and the vibration detection threshold. Person for QST assessment was blinded.

### Statistical analysis

Paired comparisons of baseline variables according to randomization were performed by the Mann–Whitney test for continuous variables and by Fisher’s exact test for categorical variables. Correlations of continuous variables were assessed by Spearman’s rank correlation coefficient. 95% confidence intervals (95% CI) were computed using 5000 bootstrap samples. The primary endpoint of the study was achievement of normal values (<10 s) in the CRT at follow-up. Based on previously reported data we assumed that 30% of patients in the standard arm will achieve a normal CRT after completion of the program. A clinically meaningful improvement was defined as a proportion of 60% of patients with normal CRT after completion of the program. To show this with an alpha of 5% and a power of 90% 122 patients had to be randomized in a 1:1 ratio. Secondary endpoints were time in seconds necessary to complete the CRT, severity of peripheral neuropathy categorized according to NCI-CTC and FACT/GOG-NTX, quality of life with global, functional and symptom score as well as global QoL, absence or presence of Achilles and patellar tendon reflexes, and all dimensions of quantitative sensory testing. Secondary endpoints were analyzed by comparing differences between baseline and subsequent assessment time points using one -sample Wilcoxon signed rank test and Mann–Whitney test. The secondary endpoints were additionally analyzed using generalized estimating equations for longitudinal assessments. With a GEE model for the CRT using a lognormal distribution for rising times in seconds and assuming a time dependency of a first order autoregressive process for the four time points was set up.

All statistical analyses were performed with the statistical software environment R, version 3.2.1, and geepack, version 1.2-0, and lsmeans, version 2.23 [34].

## Results

### Baseline characteristics

**Fig. 1:**
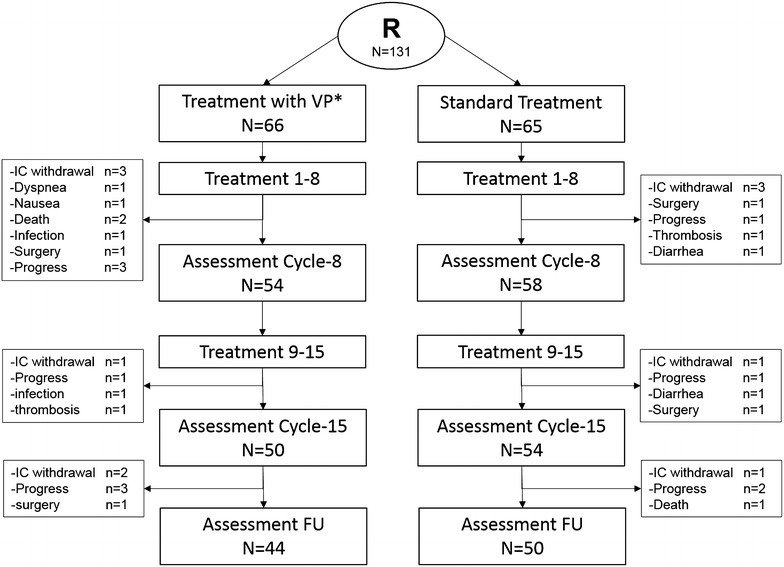
CONSORT diagram. This *figure* shows the clinical course after randomization. N = 66 patients were randomized to the experimental arm and n = 65 patients to the standard arm. N = 44 in the experimental arm and n = 50 in the standard arm reached the follow up period. *R* randomization, *IC* informed consent, *n* number, *FU* follow up, *WBV* whole-body vibration therapy

A total of 131 patients (n = 63 male, n = 68 female) were randomized into the experimental arm (n = 66 patients) and the standard arm (n = 65 patients). The CONSORT diagram is displayed in Fig. 1. The patient characteristics according to treatment arm were well balanced except time since last and time since first chemotherapy with longer time intervals in the experimental arm (Table 1). Overall median age was 60.5 years (range 24–71 years), two-thirds of the patients were diagnosed with solid tumors mostly being of stage III/IV according to WHO classification and one-third with hematological malignancies. All patients developed CIPN after intensive chemotherapy, 97% after combination chemotherapy and 35% with additional radiotherapy. According to NCI-CTC CIPN was categorized as grade III in 52% and grade II in 48%. At the time of study entry 27% (36/131) of the patients had active treatment (to control CIPN induced pain). According to FACT/GOG-NTX the patients mainly reported substantial (comprising the categories “somewhat”, “quite a bit”, “very much”) tingling as well as discomfort in the feet (97 and 98%, respectively) which corresponded to the perception of a severely low muscle mass (86%) and a general severe weakness (74%), whereas symptoms in the upper extremities were rare (Additional file 2: Table S2). The QoL assessed with the QLQ-C30 revealed before the start of any intervention a median global status of 50% (range 0–83%), a median function-related QoL of 49% (range 9–91%) a median symptom-oriented QoL of 62% (range 21–92%) and a median overall QoL of 53% (16–90%) without differences in the treatment arms. On neurological examination Achilles tendon reflexes were absent bilaterally in 58% of participants while absent patellar reflexes bilaterally in 42% without differences in the treatment arms. Quantitative sensory testing revealed a severely impaired WDT and CDT of +12.3 °C (range 1.1–18 °C) and −7.8 °C (range −32 to −1.7 °C), respectively, as well as MDT of 13.9 mN (0.2–724 mN). All patients had abnormal CRT results (≥10 s). At baseline higher WDT was in trend associated with longer time needed to complete the CRT (rho = 0.16, 95% CI = [−0.03, 0.34]), which was not the case for the CDT and MDT (rho = −0.07, 95% CI = [−0.25, 0.11] and rho = −0.06, 95% CI = [−0.24, 0.12], respectively).

**Table 1:** Patient characteristics at baseline according to treatment arm

	Treatment with WBV	Treatment without WBV	p value
	N = 66	N = 65	
Sex, male/female	29/37	34/31	0.38
Age, years, median (Range)	59 (28–70)	62 (24–71)	0.10
Diagnosis, no. (%)			0.85
Hematological malignancies	23 (35)	21(32)	
Multiple myeloma	8 (12)	11 (17)	
Lymphomas	10 (15)	7 (11)	
Leukemias	5 (7.5)	3 (5)	
Solid tumors	43 (65)	44 (68)	
Colorectal	13 (20)	12 (18.5)	
Lung	5 (7.5)	3 (4.5)	
Esophageal/gastric	2 (3)	10 (15)	
Breast/ovarian	13 (20)	12 (18.5)	
Other	7 (10.5)	4 (6)	
Disease state (excl. leukemias)			
Multiple myeloma (Salmon-Durie)			
I		3	
II	4	1	
III	4	7	
Lymphoma (Ann-Arbor)			
I	1		
II	1		
III	4	1	
IV	4	6	
Solid tumors (WHO)			
I	2	4	
II	8	10	
III	19	10	
IV	14	20	
Pretreatment, no. (%)			
Radiotherapy	28 (42%)	18 (28%)	0.10
Chemotherapy	66 (100%)	65 (100%)	
Combination chemotherapy^a^	64 (97%)	63 (97%)	0.99
Platinum-based	27 (41%)	35 (54%)	0.16
Taxane-based	22 (33%)	17 (26%)	0.45
Vinca alkaloids	9 (14%)	8 (12%)	0.99
Bortezomib	6 (9%)	7 (11%)	0.78
Time since last chemotherapy			
In months, median (range)	2 (0–98)	0 (0–51)	0.07
Time since first chemotherapy			
In months, median (range)	19 (4–156)	9 (2–180)	0.005
Treatment related neuropathy			
NCI-CTC			
Grade II	28 (42%)	35 (54%)	0.22
Grade III	38 (58%)	30 (46%)	
Active treatment to control pain, No. (%)	15 (23%)	21 (32)	0.24
Chemotherapy during study No. (%)	25 (38%)	34 (51%)	0.12

*WBV* whole-body vibration therapy, *WHO* World Health Organization, *NCI CTC* National Cancer Institute Common Toxicity Criteria

^a^Single agent chemotherapy; experimental arm, n = 2, arsenic trioxide, radioiodine therapy; standard arm, n = 2, cladribine, trastuzumab

### Treatment phase

All patients started with the first intervention, 66 in the experimental arm with WBV and 65 in the standard arm. Within the first 8 sessions 12 (experimental) and 7 (standard), terminated the program early (Fig. 1) due to withdrawal of the IC (n = 6), progress of the underlying malignant disease (n = 4), surgery (n = 2), death (n = 2) or for other reasons (n = 5). Thus 54 and 58 patients were evaluated after 8 treatment sessions. Further 8 patients, 4 in each arm, terminated the program during the following 7 treatment sessions resulting in 50 and 54 patients being evaluated after 15 treatment session. Further testing at follow-up was performed in 44 (experimental arm) and 50 (standard arm) patients, respectively. Thus 67% (44/66) and 77% (50/65) of the patients completed the whole program in the experimental and standard arm, respectively.

### Evaluation of the primary endpoint

**Fig. 2:**
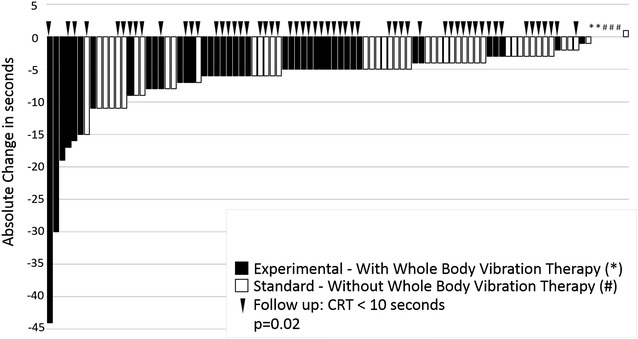
Distribution of individual absolute time-differences in seconds between baseline and follow-up needed to complete the CRT according to randomization (*black*, experimental arm with whole-body vibration therapy; *white*, standard arm). *Arrows* indicate patients with normal values for completion of the CRT at follow-up. Patients with no change were marked by a ‘*’ (experimental arm) and ‘#’ (standard arm), respectively. *CRT* chair-rising test

At baseline all patients had a CRT with pathological values with ≥10 s (median, 14 s; range 10–55 s) without significant difference in the treatment arms (p = 0.20). During the treatment course the proportion of patients with a normal CRT significantly increased to 19% (17% standard, 20% experimental) after 8 sessions, 51% (56% standard, 46% experimental) after 15 sessions and 62% (56% standard, 68% experimental) at follow-up without significant differences between the two arms at the respective time points (p = 0.40, p = 0.30, p = 0.20, respectively). Thus, in contrast to our initial sample size planning the proportion of patients with normal values to complete the CRT was higher in the standard arm as expected. However, the reduction of time needed to complete the CRT from baseline to follow-up assessment was significantly higher in the experimental arm (−5.5 s) compared to the standard arm (−4.0 s) (Fig. 2).

### Evaluation of the secondary endpoints

During treatment and at follow-up the proportion of patients with impairment in the lower extremity according the FACT/GOG-NTX categories “tingling” as well as “discomfort” in the feet were significantly (p < 0.001, p < 0.001) reduced from 97 to 81% and 98 to 71%, respectively. This was in trend more frequently observed for the category “discomfort in the feet” in the experimental arm compared to the standard arm after 15 treatment sessions and at follow-up (Additional file 2: Table S2). All other categories evaluated according the FACT/GOG-NTX indicated again a significant improvement over time but no difference between study arms. Similarly, global status, functional and symptoms score as well as overall QoL (EORTC QLQ C30) improved over time but again without differences between the study arms (Additional file 2: Table S2). The recorded walking distance per day did not increase overall with any difference between the study arms.

During treatment and at follow-up a marked improvement of the neurological reflex status in the lower extremities occurred, with consistently lower proportions of patients with absent Achilles and patellar tendon reflexes. However, this difference did not reach statistical significance (Additional file 2: Table S2).

**Fig. 3:**
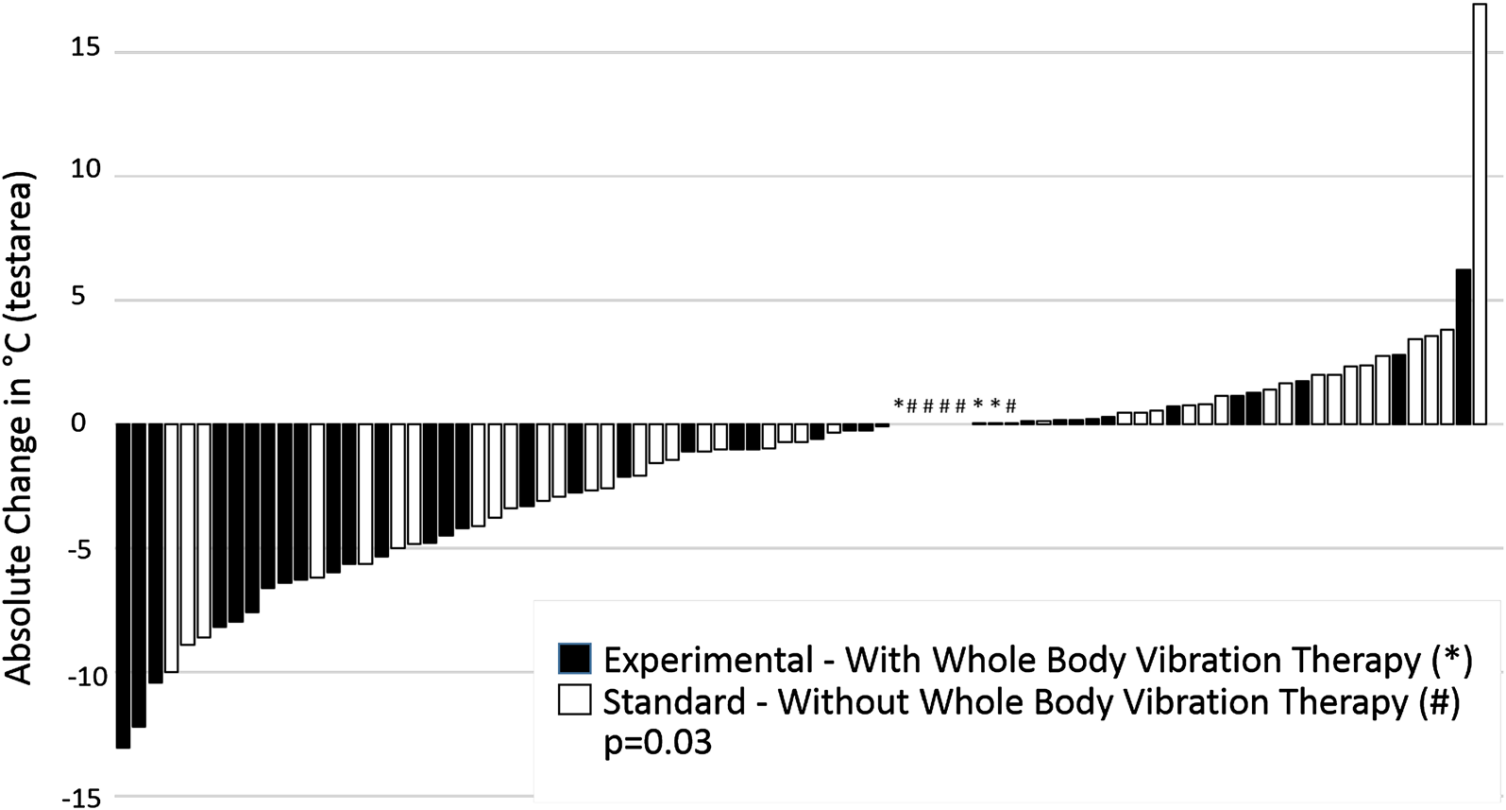
Distribution of individual absolute differences in degrees Celsius between baseline and completed program of the warm detection threshold (WDT) assessed with quantitative sensory testing according to randomization (*black*, experimental arm with whole-body vibration therapy;* white*, standard arm). Patients with no or very little change were marked by a ‘*’ (experimental arm) and ‘#’ (standard arm), respectively. *WDT* warm detection threshold

Quantitative sensory testing before the intervention and after completion of the program revealed a significant reduction in the WDT in the experimental arm compared to the standard arm (paired rank order test, p = 0.03), whereas in the other assessed qualities no relevant improvement was noted overall and between the study arms (Fig. 3, Table 2). Consistently, the GEE model for longitudinal assessments revealed that the WDT was reduced significantly over time and in particular in the experimental arm (interaction test, p = 0.04).

**Table 2:** Quantitative sensory testing over time

	Total	Treatment with WBV	Treatment without WBV	p value
C-fibers				
Warm detection threshold (WDT)				
Baseline	n = 119	n = 55	n = 64	
Test-area °C (range)	12.3 (1.1 to 18.0)	12.6 (1.3 to 18.0)	12.1 (1.1 to 18.0)	0.49
Reference-area °C (range)	1.2 (0.5 to 22.8)	1.2 (0.5 to 22.8)	1.2 (0.5 to 17.6)	0.45
After 15 Interventions	n = 87	n = 40	n = 47	
Test-area °C (range)	10.4 (1.3 to 18.0)	9.5 (1.3 to 18.0)	11.0 (2.7 to 18.0)	0.09
Reference-area °C (range)	1.3 (0.5 to 15.8)	1.3 (0.5 to 8.8)	1.3 (0.5 to 15.8)	0.72
Difference	n = 87	n = 40	n = 47	
Test-area Δ °C (range)	−0.7 (−13.1 to 16.9)	−1.1 (−13.1 to 6.2)	−0.1 (−10.0 to 16.9)	0.03
Reference-area Δ °C (range)	0.0 (−22.2 to 2.2)	0.0 (−22.2 to 2.2)	0.0 (−16.5 to 2.0)	0.35
Heat pain threshold (HPT)				
Baseline	n = 129	n = 55	n = 64	
Test-area °C (range)	48.6 (34.9 to 50.0)	48.7 (34.9 to 50.0)	48.3 (39.2 to 50.0)	0.39
Reference-area °C (range)	44.7 (33.8 to 50.0)	44.7 (33.9 to 49.5)	44.7 (33.8 to 50.0)	0.36
After 15 Interventions	n = 87	n = 40	n = 47	
Test-area °C (range)	48.5 (37.4 to 50.0)	48.1 (37.4 to 50.0)	48.7 (39.9 to 50.0)	0.47
Reference-area °C (range)	44.9 (33.8 to 50.0)	44.8 (33.9 to 50.0)	45.2 (33.8 to 50.0)	0.48
Difference	n = 85	n = 38	n = 47	
Test-area Δ °C (range)	0.0 (−7.2 to 8.94)	−0.04 (−3.93 to 3.57)	0.0 (−3.34 to 3.60)	0.54
Reference-area Δ °C (range)	0.0 (−4.4 to 0.6)	0.0 (−0.12 to 4.23)	0.0 (−0.51 to 2.12)	0.92
Aδ-fibers				
Cold detection threshold (CDT)				
Baseline	n = 110	n = 56	n = 64	
Test-area °C (range)	−7.8 (−32.0 to 1.7)	−7.6 (−32.0 to −1.7)	−8.4 (−32.0 to −1.9)	0.35
Reference-area °C (range)	−1.2 (−21.9 to 0.5)	−1.1 (−9.7 to −0.5)	−1.4 (-21.9 to −0.5)	0.08
After 15 Interventions	n = 88	n = 41	n = 47	
Test-area °C (range)	−6.8 (−32.0 to −1.3)	−6.6 (−32.0 to −1.3)	−6.9 (−32.0 to −1.6)	0.36
Reference-area °C (range)	−1.3 (−21.9 to 0.5)	−1.1 (−6.8 to −0.47)	−1.4 (−21.9 to −0.5)	0.12
Difference	n = 85	n = 38	n = 47	
Test-area Δ °C (range)	0.7 (−11.1 to 26.9)	1.1 (−10.1 to 24.9)	0.7 (−11.1 to 26.9)	0.46
Reference-area Δ °C (range)	0.0 (−4.0 to 6.7)	0.0 −0.3 to 6.7)	0.0 (4.0 to 3.9)	0.23
Aβ-fibers				
Mechanical detection threshold (MDT)				
Baseline	n = 110	n = 56	n = 64	
Test-area mN (range)	13.9 (0.2 to 724)	12.6 (0.9 to 724)	16.6 (0.2 to 223)	0.44
Reference-area mN (range)	0.2 (0.2 to 6.1)	0.2 (0.2 to 6.1)	0.2 (0.2 to 4.6)	0.20
After 15 Interventions	n = 88	n = 41	n = 47	
Test-area mN (range)	17.8 (0.3 to 724)	19.7 (0.3 to 724)	17.2 (1.3 to 724)	0.73
Reference-area mN (range)	0.2 (0.2 to 11.3)	0.2 (0.2 to 11.3)	0.2 (0.2 to 4.6)	0.26
Difference	n = 85	n = 38	n = 47	
Test-area mN (range)	0.0 (−174 to 501)	0.8 (−174 to 207)	−3.4 (−105 to 501)	0.35
Reference-area mN (range)	0.0 (−0.5 to 4.8)	0.0 (−0.02 to 4.8)	0.0 (−0.5 to 0.2)	0.94

*WBV* whole-body vibration therapy, *°c* degree Celsius, *mN* Millinewton, *WDT* warm detection threshold, *CDT* cold detection threshold, *HPT* heat pain threshold, *MDT* mechanical detection threshold

### Multivariable analysis for longitudinal assessments

**Fig. 4:**
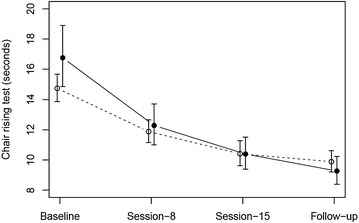
Predicted times and their 95% confidence intervals for completion of the CRT according to a GEE model including assessment time points (baseline, 8th session, 15th session, follow-up), treatment arm (*open circle*, *dashed line*, without WBV; *filled circle*, *solid line*, with WBV) and initial warm detection threshold (WDT). Predictions are shown given the overall mean WDT level of 11.66. *CRT* chair-rising test, *GEE* generalized estimated equation, *WBV* whole-body vibration therapy, *WDT* warm detection threshold

An exploratory analysis on the time needed to complete the CRT measured in seconds including all assessments from baseline to follow-up, treatment arm and initial WDT using a GEE model for longitudinal assessments revealed that a baseline higher WDT was associated with a longer time needed for the CRT over time (estimate, 0.015, standard error [SE], 0.006, p = 0.02), that the time needed for the CRT decreased significantly over time (p < 0.001), and that patients treated in experimental arm had a significantly higher reduction in the time needed for the CRT compared to those treated in standard arm (interaction test, p = 0.03), (Fig. 4).

## Discussion

The aim of our study was to evaluate the relative impact of WBV within a structured training program to treat CIPN. Overall, the addition of WBV resulted in a significant reduction in time needed to complete the CRT (Fig. 2) indicating better fitness and balance as well as a significant reduced WDT (Fig. 3) as read out for improved sensory function after completion of the program.

All 131 patients were treated with massage, mobilization as well as physical exercises and were randomized for the addition of WBV (n = 66) during biweekly treatment sessions. N = 94 reached follow up after 15 treatment sessions. As primary endpoint we used the CRT as a surrogate marker for physical fitness and posture balance; secondary endpoints were Qol, the FACT/GOG-NTX, blinded quantitative sensory testing and physical examination. Based on a recently published meta-analysis a prevalence of CIPN as high as 68% in the first month after chemotherapy was reported with a resolution of symptoms in only 12% of the affected patients after 3 months [1] and persistence of symptoms up to 11 years after chemotherapy [35]. In contrast, in our study all patients experienced marked improvement in physical fitness and posture balance with more than 50% of the patients with a normal CRT, a marked improvement in quality of life based on symptoms and function scores assessed by EORTC QLQ-C30 as well as an improved tendon reflex status. However, still most of our patients suffered from tingling and discomfort in the feet as measured with the FACT/GOG-NTX. Although our study was not randomized versus observation, the overall results suggest an important beneficial impact of massage, mobilization as well as physical exercises in the management of CIPN. Furthermore, WBV applied in addition to this program seems to further improve outcome with in trend less patients suffering from discomfort in the feet, significantly better performance in the CRT and an improved WDT after completion of all training sessions. Compared to attempts with medical therapy using anti-epileptics or antidepressants [15–18], anti-oxidative and other neuroprotective drugs [11, 12], as well as herbal medicine [13], our approach showed effects measured by objective endpoints including the CRT and the WDT as well as in subjective endpoints measured by the FACT/GOG-NTX. Interestingly, electroacupuncture also showed an improvement of CIPN measured with the FACT/GOG-NTX scale [14]. Due to the heterogeneity of applied chemotherapeutic agents and their combinations as well as limited sample size, we were not able to perform meaningful subset-analyses for selected agents or combinations. However, in our initial sample size calculation we underestimated the effect of massage, mobilization as well as physical exercises alone with a normalized CRT in 56% of our patients after completion of the program in the standard arm and thus we were not able to show the initially projected improvement. Nevertheless, the reported results showed an improvement in objective and subjective endpoints. CIPN affects mostly large Aβ myelinated fibers and unmyelinated C fibers [36]. The function of the unmyelinated C-fibers can be estimated by use of the WDT [33]. By the addition of WBV, we observed a reduction of the WDT by in median 1.1 °C in the experimental arm compared to 0.1 °C (p = 0.03) in the standard arm (Table 2). To our knowledge this is the first time such a finding has been described. A higher WDT was associated with a longer time needed for the CRT and significantly impacted the time needed for the CRT in our multivariable model with repeated measurements. Thus the WDT may be an early read-out of an improvement in CIPN.

In conclusion, the treatment of CIPN with a program including massage, mobilization as well as physical exercises and WBV had a significantly and clinically relevant beneficial impact on symptoms relieve, physical fitness and sensory function. The integration of this program into daily clinical practice is desirable but will require a structured prospective assessment of CIPN and importantly a specialized education of nursing staff.

## Additional files

**Additional file 1: Table S1.** Exercise description.

**Additional file 2: Table S2.** Test results for FACT/GOG Ntx, quality of life (Qol) and tendon reflex status.

## Abbreviations

CDT: cold detection threshold
CIPN: chemotherapy-induced polyneuropathy
CPT: cold pain threshold
CRT: chair-rising test
FACT/GOG-NTX: Functional Assessment of Cancer Therapy/Gynecologic Oncology Group neurotoxicity subscale
FU: follow up
GEE: generalized estimated equation
HPT: heat pain threshold
IC: informed consent
IP: integrated program
MDT: mechanical detection threshold
MPS: mechanical pain sensitivity
MPT: mechanical pain threshold
n: number
NCI CTC: National Cancer Institute Common Toxicity Criteria
PPT: pressure pain threshold
QoL: quality of life
QST: quantitative sensory testing
R: randomization
SE: standard error
TSL: thermal sensory limen
WBV: whole-body vibration therapy
WDT: warm detection threshold
WHO: World Health Organization
WUR: wind-up ratio
