# Supplementary material for: A randomized exploratory phase 2 study in patients with chemotherapy-related peripheral neuropathy evaluating whole-body vibration training as adjunct to an integrated program including massage, passive mobilization and physical exercises
Source: Exp Hematol Oncol. 2017 Feb 7;6:5. doi: 10.1186/s40164-017-0065-6 (PMC5297221; doi:10.1186/s40164-017-0065-6)
Supplement: Supplementary file 1 — Additional file 1: Table S1. Exercise description. [file 40164_2017_65_MOESM1_ESM.docx]

### Supplementary Table 1. Exercise description

| 1. | Foot rotation in supine position (3 times, each) |
| --- | --- |
| 2. | Sit-ups-1: movement from supine position to seated forward bend position (3 times) |
| 3. | Supine position, shorten and lengthen legs circularly (3 times, each direction) |
| 4. | From supine position to shoulder bridge (3 times) |
| 5. | Supine bound angle pose (3 times) |
| 6. | Leg elevation and lowering from supine position (3 times) |
| 7. | Leg rotation over the pelvis in supine position with bended legs (3 times) |
| 8. | Sit-ups-2, lying face up with raised legs (5 times) |
| 9. | Lying on the back with raised legs at a 90° angle to the ceiling, lifting of the pelvis with external rotation in both legs (5 times) |
| 10. | Trunk rotation lying face up with raised legs, head and chest turns in the opposite direction from legs and pelvis (5 times) |
| 11. | Lying face up with legs raised, moving arms circularly along the floor and over the body, alternating start with internal or external rotation (3 times) |
| 12. | Lying face up with raised legs, shoulder blade mobilization (5 times) |
| 13. | Lying face up with raised legs. Functional integration of shoulder and arm movement |
| 14. | Side leg lift combined with alternately lift and lower from the thorax (10 times, each) |
| 15. | Trunk rotation in side position (5 times, each side) |
| 16. | Alternating leg lift in prone position (5 times, each side) |
| 17. | Rotation of the lower legs in prone position. Alternating of internal and external rotation (3 times, each direction) |
| 18. | Modified push-ups: movement from “child pose” to “cobra pose” (3 times) |
| 19. | Tiptoeing (10 times) |
| 20. | Standing up in a parallel movement (3 times) |
| 21. | Standing up in a helical movement (3 times, each direction) |
