# Supplementary material for: A randomized exploratory phase 2 study in patients with chemotherapy-related peripheral neuropathy evaluating whole-body vibration training as adjunct to an integrated program including massage, passive mobilization and physical exercises
Source: Exp Hematol Oncol. 2017 Feb 7;6:5. doi: 10.1186/s40164-017-0065-6 (PMC5297221; doi:10.1186/s40164-017-0065-6)
Supplement: Supplementary file 2 — Additional file 2: Table S2. Test results for FACT/GOG Ntx, quality of life (Qol) and tendon reflex status. [file 40164_2017_65_MOESM2_ESM.docx]

### Supplementary Table 2. Test Results for FACT/GOG Ntx, Quality of life (Qol) and Tendon reflex status

|  | Total | Treatment with WBV | Treatment without WBV | p-value |
| --- | --- | --- | --- | --- |
| Baseline |  |  |  |  |
| No. | 131 | 66 | 65 |  |
|  | No. (%) | No. (%) | No. (%) |  |
| Tingling in the feet* | 127 (97) | 62 (94) | 65 (100) | 0.12 |
| Discomfort in the feet* | 128 (98) | 63 (95) | 65 (100) | 0.24 |
| Low muscle mass* | 111 (85) | 56 (85) | 55 (85) | 0.99 |
| General weakness* | 97 (74) | 50 (76) | 47 (72) | 0.69 |
|  |  |  |  |  |
| After 8 Interventions |  |  |  |  |
| No. | 111 | 54 | 57 |  |
|  | No. (%) | No. (%) | No. (%) |  |
| Tingling in the feet* | 104 (94) | 50 (93) | 54 (95) | 0.71 |
| Discomfort in the feet* | 99 (89) | 47 (87) | 52 (91) | 0.55 |
| Low muscle mass* | 64 (58) | 28 (52) | 36 (64) | 0.25 |
| General weakness* | 57 (51) | 28 (52) | 29 (51) | 0.99 |
|  |  |  |  |  |
| After 15 Interventions |  |  |  |  |
| No. | 102 | 49 | 53 |  |
|  | No. (%) | No. (%) | No. (%) |  |
| Tingling in the feet* | 83 (81) | 37 (76) | 46 (87) | 0.20 |
| Discomfort in the feet* | 75 (74) | 32 (65) | 43 (81) | 0.08 |
| Low muscle mass* | 36 (35) | 14 (29) | 22 (42) | 0.21 |
| General weakness* | 30 (29) | 13 (26) | 17 (32) | 0.66 |
|  |  |  |  |  |
| Follow-up |  |  |  |  |
| No. | 92 | 44 | 48 |  |
|  | No. (%) | No. (%) | No. (%) |  |
| Tingling in the feet* | 75 (81) | 34 (77) | 41 (87) | 0.27 |
| Discomfort in the feet* | 65 (71) | 27 (61) | 38 (79) | 0.07 |
| Low muscle mass* | 35 (38) | 15 (34) | 20 (42) | 0.52 |
| General weakness* | 30 (33) | 16 (36) | 14 (30) | 0.66 |
|  |  |  |  |  |
| **comprises the extensions “somewhat”, “quite a bit”, “very much”* | | | | |
|  |  |  |  |  |
| Quality of Life - QLQ-C30 (in %) | | | | |
|  |  |  |  |  |
| Baseline |  |  |  |  |
| No. | 129 | 65 | 64 |  |
|  | median (range) | median (range) | median (range) | p-value |
| Global status | 50 (0-83) | 42 (0-83) | 50 (0-83) | 0.84 |
| Symptoms score | 62 (21-92) | 62 (26-92) | 62 (21-92) | 0.85 |
| Function score | 49 (9-91) | 49 (9-91) | 53 (13-91) | 0.81 |
| Overall QoL | 53 (16-90 | 53 (16-90) | 56 (21-86) | 0.77 |
|  |  |  |  |  |
| After 8 Interventions |  |  |  |  |
| No. | 108 | 52 | 56 |  |
| Global status | 50 (0-83) | 50 (17-83) | 50 (0-83) | 0.81 |
| Symptoms score | 72 (31-100) | 72 (41-95) | 71 (31-100) | 0.47 |
| Function score | 65 (11-98) | 67 (13-96) | 62 (11-98) | 0.99 |
| Overall QoL | 68 (28-96) | 69 (28-93) | 67 (29-96) | 0.67 |
|  |  |  |  |  |
| After 15 Interventions |  |  |  |  |
| No. | 100 | 48 | 52 |  |
| Global status | 67 (17-92) | 67 (17-92) | 50% (17-83) | 0.38 |
| Symptoms score | 79 (31-100) | 79 (38-100) | 77 (31-100) | 0.63 |
| Function score | 73 (20-100) | 73 (22-100) | 70 (20-100) | 0.82 |
| Overall QoL | 73 (31-99) | 77 (31-99) | 72 (34-98) | 0.73 |
|  |  |  |  |  |
| Follow-up |  |  |  |  |
| No. | 92 | 44 | 48 |  |
| Global status | 67 (8-100) | 67 (8-100) | 58 (17-83) | 0.37 |
| Symptoms score | 82 (15-100) | 83 (36-100) | 82 (15-100) | 0.96 |
| Function score | 73 (20-100) | 76 (31-98) | 72 (29-100) | 0.77 |
| Overall QoL | 77 (34-98) | 79 (39-98) | 73 (34-98) | 0.89 |
|  |  |  |  |  |
| Tendon reflex status - bilaterally absent | | | | |
|  |  |  |  |  |
| Baseline |  |  |  |  |
| No. | 130 | 65 | 65 |  |
| Achilles tendon reflex | 75 (58%) | 35 (53%) | 40 (61%) | 0.48 |
| patellar tendon reflex | 54 (42%) | 21 (38%) | 33 (51%) | 0.20 |
|  |  |  |  |  |
| After 8 Interventions |  |  |  |  |
| No. | 102 | 50 | 52 |  |
| Achilles tendon reflex | 39 (38%) | 18 (36%) | 21 (40%) | 0.69 |
| patellar tendon reflex | 46 (45%) | 20 (40%) | 26 (50%) | 0.33 |
|  |  |  |  |  |
| After 15 Interventions |  |  |  |  |
| No. | 99 | 48 | 51 |  |
| Achilles tendon reflex | 34 (34%) | 13 (27%) | 21 (41%) | 0.20 |
| patellar tendon reflex | 49 (54%) | 21 (44%) | 28 (55%) | 0.32 |
|  |  |  |  |  |
| Follow-up |  |  |  |  |
| No. | 90 | 43 | 47 |  |
| Achilles tendon reflex | 25 (28%) | 9 (21%) | 16 (34%) | 0.24 |
| patellar tendon reflex | 25 (28%) | 7 (16%) | 18 (38%) | 0.03 |
| *Abbreviations: FACT/GOG: Functional Assessment of Cancer Therapy/Gynecologic Oncology Group; No.: number; QoL: quality of life; WBV: whole-body vibration therapy* | | | | |
